# Demonstration of early efficacy results of the delayed-release combination of doxylamine-pyridoxine for the treatment of nausea and vomiting of pregnancy

**DOI:** 10.1186/s12884-016-1172-9

**Published:** 2016-11-24

**Authors:** Gideon Koren, Shannon Clark, Gary D. V. Hankins, Steve N. Caritis, Jason G. Umans, Menachem Miodovnik, Donald R. Mattison, Ilan Matok

**Affiliations:** 1 University of Toronto, Toronto, Canada; 2 University of Western Ontario, London Ontario, Canada; 3 Department of Obstetrics and Gynecology, University of Texas, Medical Branch Galveston, Galveston, TX USA; 4 Department of Obstetrics and Gynecology, University of Pittsburgh, Medical Center, Pittsburgh, PA USA; 5 Medstar Health Research Institute, Hyattsville, MD USA; 6 The Georgetown- Howard Universities Center for Clinical and Translational Science, Washington DC, USA; 7 The Obstetric Pharmacology Research Unit Network, Eunice Kennedy Shriver, National Institute of Child and Human Development, Bethesda, MD USA; 8 Division of Clinical Pharmacy, Institute of Drug Research, School of Pharmacy, Faculty of Medicine, The Hebrew University of Jerusalem, Jerusalem, Israel

## Abstract

**Background:**

Nausea and vomiting of pregnancy (NVP) affects up to 80% of expecting mothers. In April 2013 the FDA approved the delayed-release combination of doxylamine succinate and pyridoxine hydrochloride (Diclegis®) for NVP, based in part, on the results of a phase III randomized trial demonstrating the efficacy of this drug combination [study drug marketed under the trade name Diclectin® in Canada and Diclegis® in the United States] compared to placebo in pregnant women. Study drug dosing occurred for 14 days, which is substantially longer than what has been performed in similar studies. The objective of this study was to evaluate, through secondary analysis, whether the primary measure of efficacy can be demonstrated after five days of treatment.

**Methods:**

Women suffering from NVP were randomized to receive Diclegis® (*n* = 131) or placebo (*n* = 125) for 14 days at doses ranging from two to four tablets a day, based on a pre-specified titration protocol. The primary efficacy endpoint was the change in the validated Pregnancy-Unique Quantification of Emesis (PUQE) score at baseline versus Day 15 between Diclegis®-treated and placebo-treated women.

For the present study, the change in PUQE score between baseline and Day 15 (end of the study) was compared to the changes observed for Days 3, 4, and 5.

**Results:**

The use of delayed-release doxylamine succinate and pyridoxine hydrochloride tablets show improved NVP symptom control as compared to placebo on Days 3,4 and 5, with sustained efficacy until the end of the trial.

**Conclusion:**

A four day study drug dosing trial with Diclegis® is sufficient to document efficacy, as the results are similar to those achieved after 14 study drug dosing days.

The benefit seen at the earlier time validates drug efficacy and minimizes the natural course of improvement.

**Trial registration:**

CTR No. NCT006 14445 2007.

## Background

Nausea and vomiting of pregnancy (NVP) affects up to 80% of expecting mothers, and for many women, pharmacotherapy is necessary to control their symptoms [1–4]. The delayed--release combination of doxylamine succinate and pyridoxine hydrochloride has been extensively studied with publications in the peer-reviewed medical literature corroborating the safe and effective use of this agent [1, 5–12].

The delayed-release characteristic of this combination allows for the starting dose of two tablets at bedtime to be effective five to seven hours later, in the morning hours when NVP symptoms tend to be most prevalent [13, 14]. In 2013, the FDA approved the sale of Diclegis®, based, in part, on results from a randomized double-blind, placebo-controlled trial [15, 16]. In this trial, pregnant women between 7 and 14 weeks’ gestation received study drug, Diclegis® or placebo, for 14 days. The NVP-specific and validated Pregnancy-Unique Quantification of Emesis (PUQE) score was collected daily; however, the *a priori* primary effectiveness endpoint was determined to be the change in NVP symptoms as measured by the PUQE score from baseline to Day 15. This period of study drug dosing for two weeks is substantially longer than other therapeutic studies in NVP which rarely continue beyond five days [17–19]. For ethical reasons, denying women suffering from NVP a safe and effective treatment, and randomizing them to placebo should be for as limited duration as possible. As such, the present secondary analysis was conducted to determine whether shorter drug dosing days would yield similar efficacy results.

## Methods

This is a secondary analysis of a double-blind, randomized, multicenter, placebo-controlled study of the delayed-release combination of doxylamine succinate (10 mg) and pyridoxine hydrochloride (10 mg) (Diclegis®) for the treatment of NVP]. The full details of the study have been previously published [15]. Briefly, the subjects were at least 18 years of age, pregnant in the gestational age range of 7–14 weeks, suffered from NVP, and had a PUQE score ≥6 [20–22]. The PUQE score incorporates the number of daily vomiting episodes, number of daily retching, and length of daily nausea in hours, for an overall score of symptoms rated from 3 (no symptoms) to 15 (most severe). Score of 1 on each of the 3 symptoms-nausea, vomiting and retching, denotes “no symptoms”, and goes as high as 15 (5, or maximum for each symptom). Scores of 4–6 denote mild NVP. Scores of 7–11 denote moderate NVP, and scores of 12–15 denote severe NVP.

Between 2-4 tablets of study drug (Diclegis® or placebo) were administered, based on severity of symptoms. The study had a 15 day period consisting of 14 dosing days. Subjects completed the PUQE score and the study diary once daily.

The primary therapeutic effect of Diclegis® or placebo was calculated by subtracting the PUQE score at baseline (ie. before receiving study drug) from the PUQE score at Day 15 (or at early termination). The difference in the change in PUQE between Diclegis®-treated and placebo-treated women was used as the primary efficacy endpoint.

For the purpose of this secondary analysis, the same calculations were conducted, but this time comparing the change in PUQE score from baseline to Day 3, from baseline to Day 4, and from baseline to Day 5 between Diclegis®-treated and placebo-treated women. Student’s *t* test for pair data was used for these comparisons.

Subsequently, a mixed model for repeated measures was used to analyse PUQE scores on days 3, 4, 5, and 15, using the ITT-E population. The change from baseline in PUQE score was the dependent variable, and treatment, study day, and treatment by study day interaction were included as categorical fixed effects, and the baseline score was included as a continuous fixed effect. The correlation between measures on different days within the same subject was modeled with an unstructured covariance matrix. Least squares means for the change from baseline at each treatment and timepoint were calculated from the model, along with the estimated treatment effect (Diclegis minus placebo) at each timepoint and its 95% confidence interval and *P* value.

## Results

A total of 131 women in the Diclegis®-treated group and 125 receiving placebo were available for analysis. The two groups did not differ in any demographic or medical characteristics [15].

As previously published, the use of Diclegis® for 14 days resulted in a significantly lower PUQE score by 0.9 PUQE units when compared to placebo (*p* = 0.006) [15]. At Day 3, the mean difference in NVP symptoms between Diclegis®-treated and placebo-treated women was also significantly lower by 1.0 PUQE units (*p* = 0.002). At Day 4, the mean difference in NVP symptoms between Diclegis®-treated and placebo-treated women was 1.1 PUQE units (*p* < 0.001). At Day 5, the mean difference was 1.0 PUQE units (*p* = 0.006) (Table 1).

**Table 1:** Efficacy of Diclegis® compared to placebo for the treatment of NVP

Study Day	Statistics	PUQE score (mean ± SD) of Diclegis®-treated women	PUQE score (mean ± SD) Placebo-treated women	*P*-value
Baseline		9.0 ± 2.1	8.8 ± 2.1	
Day 3	N	128	123	
	Day 3 PUQE score	5.9 ± 2.4	6.7 ± 2.2	
	Change from Baseline	−3.1 ± 2.7	–2.1 ± 2.4	0.002
	% Change from Baseline	34.4%	23.9%	
Day 4	N	125	120	
	Day 4 PUQE score	5.4 ± 2.2	6.3 ± 2.3	
	Change from Baseline	–3.6 ± 2.5	–2.5 ± 2.2	<0.001
	% Change from Baseline	40.0%	28.4%	
Day 5	N	122	112	
	Day 5 PUQE score	5.1 ± 2.2	5.8 ± 2.1	
	Change from Baseline	–4.0 ± 2.5	–3.0 ± 2.4	0.006
	% Change from Baseline	44.4%	34.0%	
Day 15	N	131	125	
	Day 15 PUQE score	4.2 ± 1.9	4.9 ± 2.3	
	Change from Baseline	–4.8 ± 2.7	–3.9 ± 2.6	0.006
	% Change from Baseline	53.0%	44.0%	

Change in Pregnancy-Unique Quantification of Emesis score from baseline to Days 3, 4, 5 and 15

The mixed model for repeated measureseffects shows that the effect of the treatment remained constant over time with no differences among Day 3, 4, or 5 (Table 2). By Day 15 the effect has become somewhat smaller and non significant.

**Table 2:** A mixed model for repeated measures showing PUQE scores on days 3, 4, 5, and 15, using the ITT-E population

Nature of statistic	*N*	Diclegis LS Mean	Placebo LS Mean	Estimated treatment effect (Diclegis minus placebo)	95% confidence interval	*P* value
Number of Observations Used	920					
Number of Subjects Included in Analysis	252					
Overall Tests of Fixed Effects						
TRT						0.0004
DAY						<.0001
TRT*DAY						0.3374
BASELINE						<.0001
Treatment Effects by Day						
Day 3		–3.03	–2.15	–0.88	–1.43 to–0.33	0.0019
Day 4		–3.45	–2.51	–0.95	–1.48 to–0.41	0.0006
Day 5		–3.81	–3.05	–0.76	–1.29 to–0.24	0.0045
Day 15		–5.00	–4.60	–0.40	–0.86 to 0.06	0.0867

*LS Mean* Least Squares Mean

The change from baseline in PUQE score was the dependent variable, and treatment, study day, and treatment by study day interaction were included as categorical fixed effects, and the baseline score was included as a continuous fixed effect. The correlation between measures on different days within the same subject was modeled with an unstructured covariance matrix. Least squares means for the change from baseline at each treatment and timepoint were calculated from the model, along with the estimated treatment effect (Diclegis minus placebo) at each timepoint and its 95% confidence interval and *P* value

Overall, the difference in efficacy between Diclegis® and placebo remained quite stable and the results on Day 4 showed a slightly larger impact than in the other days. Represented as a percentage change from baseline, on Day 4, the Diclegis®-treated group had a 40% change from baseline PUQE score compared to a 28.4% change from baseline PUQE score for the placebo-treated group (Table 1).

## Discussion

The randomized trial described herein has previously shown the superiority of Diclegis® over placebo in treating symptoms of NVP in American women [15] as well as its maternal safety [23]. The study had a placebo arm, as symptoms of NVP may subside spontaneously in women by the end of the first trimester [3, 4, 24], and without a placebo arm the improvement may be erroneously and solely attributed to the active drug. However, in clinical practice it is important to note that over 20% of women experience symptoms beyond the first trimester of pregnancy [3, 4, 24].

In the present analysis, the greatest difference in the change in PUQE score between Diclegis®-treated and placebo-treated women remained stable on Days 3,4,5; by Day 15, some efficacy attributed to placebo may have been the result of spontaneous resolution of symptoms, causing the effect to loose its statistical significance. The results of the present analysis demonstrate that having a shorter duration trial of four or five days to determine the efficacy of Diclegis® may be favourable as compared to longer duration trials.

Continuation of a trial for 14 days when women with NVP are randomized to placebo may result in a substantial decreased quality of life; therefore, shorter duration trials are ideal when possible. This is especially true for a special population such as pregnant women. Additionally, a longer trial necessitates careful follow up of patients’ adherence, as there is a tendency toward less compliance over time as well as loss to follow-up.

In this study, a secondary analysis was conducted on Diclegis® efficacy as compared to placebo after three, four or five days of study drug dosing, as compared to the original study that conducted data analysis to assess efficacy after 14 dosing days. Most studies conducted to-date on efficacy of antiemetics for NVP were much shorter, typically three to five days long but no standardized duration of measurement is in place [17–19]. A standardized duration of measurement would also allow for a more accurate comparison of results across studies. Based on this secondary analysis of results from the 14 day long Phase III double-blind placebo controlled trial, the therapeutic response could be established earlier.

The benefit seen at the earlier time validates drug efficacy and minimizes the natural course of improvement. These results can guide the design of future studies comparing the effectiveness of the doxylamine and pyridoxine combination, and in turn, improving the quality of life of study participants.

## Conclusions

A four day study with Diclegis® is sufficient to document efficacy, as the results are similar to those achieved after 14 study drug dosing days.

## Abbreviations

ACOG: American College of obstetrics and gynecology
AE: Adverse events
ANCOVA: Analysis of covariance
FDA: Food and drug administration
NVP: Nausea and vomiting of pregnancy
PUQE: Pregnancy-unique quantification of emesis
